# Epidemiology and Clinical Management of Rheumatic Autoimmune Diseases in the COVID-19 Pandemic: A Review

**DOI:** 10.3389/fmed.2021.725226

**Published:** 2021-08-19

**Authors:** Yingzi Zhu, Jixin Zhong, Lingli Dong

**Affiliations:** Department of Rheumatology and Immunology, Tongji Hospital, Tongji Medical College, Huazhong University of Science and Technology, Wuhan, China

**Keywords:** rheumatic autoimmune diseases, COVID-19, prevalence, treatment, laboratory indicator

## Abstract

The coronavirus disease 2019 (COVID-19) has been in pandemic for more than 1 year, with serious negative effects produced worldwide. During this period, there have been a lot of studies on rheumatic autoimmune diseases (RADs) combined with COVID-19. The purpose of this study is to review and summarize these experiences. Pubmed, Web of science, Embase and the Cochrane library were searched from January 15, 2020 to July 15, 2021 using RADs and COVID-19 related keywords. Based on a comprehensive review of studies covering 16 countries, the prevalence of COVID-19 does not necessarily increase in RADs patients compared to the general population. In RADs population infected with COVID-19, a high proportion of female patients (54.44~95.2%), elderly patients (≥50y, 48~75.88%), and patients with pre-existing comorbidities (respiratory, 4.8~60.4%; endocrine, 8.52~44.72%; cardiovascular, 15.7~64.73%) were observed, although, this does not appear to have a decisive effect on disease severity. Many anti-rheumatic treatments have been extensively evaluated for their efficacy of treating COVID-19 in RADs patients, with TNF-α inhibitors and IL-6 receptor antagonist receiving more positive reviews. However, there is no conclusive information for most of the therapeutic regimens due to the lack of high-level evidence. Inflammatory markers or neutrophil-lymphocyte-ratio may be applied as indicators for clinical prognosis or therapeutic regimens adjustment. Thus, more research is still needed to address the prevalence, treatment, and clinical monitoring of RADs patients in COVID-19 pandemic.

## Introduction

Since the end of 2019, the outbreak of coronavirus disease 2019 (COVID-19) has caused great consternation worldwide. Over the past year, rheumatologists have been actively working on the impact of COVID-19 on rheumatic autoimmune diseases (RADs) patients, and this topic is still on going. In the early stage of the pandemic, many studies suggested an increased incidence of COVID-19 in RADs patients (1–3). However, along with more clinical cases were included, different opinions have arisen. Meanwhile, a growing body of research has investigated the influencing factors of COVID-19 infection in RADs patients (4–6). Besides, the most important but controversial issue is the treatment for COVID-19 infection in RADs patients. Whether the presence of COVID-19 aggravates the existing RAD, or the underlying RAD increases the susceptibility of patients to COVID-19, establishing an effective therapeutic regime is a huge challenge. Several studies have suggested potential treatments, although, their benefits are limited to certain clinical conditions (7–10). It is therefore, particularly important to sum up the lessons learned during the pandemic.

Herein, we searched Pubmed, Web of science, Embase and the Cochrane library databases with related keywords, including “coronavirus disease 2019,” “COVID-19,” “SARS-CoV-2,” “rheumatic immune diseases,” “rheumatoid arthritis,” “systemic lupus erythematosus,” “Sjogren Syndrome,” “sicca,” “Inflammatory myopathy,” and “IgG4-related disease.” The cutoff point for search is July 15, 2021. The inclusion criteria are: (1) The content of article involves the incidence, mortality or hospitalization rates of COVID-19 in RADs patients, the risk factors, treatment, and laboratory indicators; (2) Retrospective or prospective studies written in English, the full text of which is available online. The exclusion criteria are: (1) Short articles with incomplete information; (2) Single case report. After a detailed review, a series of epidemiological and clinical management characteristics of COVID-19 infection in RADs patients were obtained.

## Epidemiological Features

**Figure 1:**
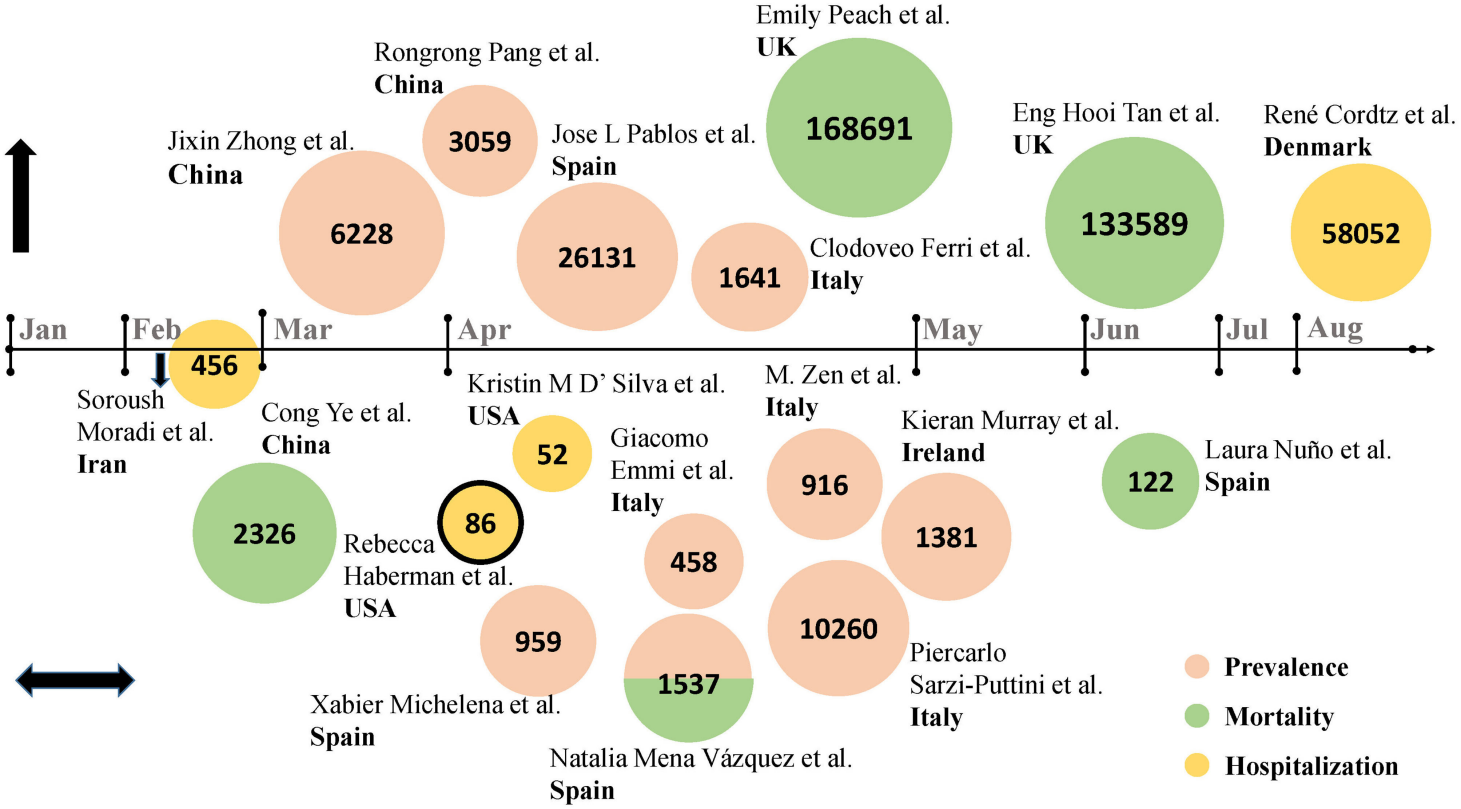
Studies from different countries on the epidemiology of COVID-19 in RADs patients (Data from Table 1). Eighteen studies were included. Authors and country were detailed in the figure. The numbers in the circles represent sample size. The nodes on the horizontal axis represent the corresponding month, above the horizontal axis are studies that reported an increasing trend in indicators in RADs patients with COVID-19, below are studies that reported a similar trend in indicators, on the horizontal axis is study that reported a downward trend in indicators. Pink circles represent prevalence, green circles represent mortality, and yellow circles represent hospitalization rates. The combination of red and green indicates that the study reported both morbidity and mortality. The circle with black outline represents case series, and the rest are retrospective cohort studies.

First, in terms of incidence, hospitalization rate and mortality, multiple studies were sorted out and presented in the order of cutoff date (Figure 1, data extracted from Table 1). The number of studies increased significantly in late March and throughout April, when the epidemic was going through a phase of surge in cases. Initially, as the place where the severe acute respiratory syndrome coronavirus 2 (SARS-CoV-2) was first detected, a multi-center retrospective study in Hubei province revealed a higher diagnostic rate of COVID-19 in RADs patients than in their family members without RADs (2). In Spain, data from seven hospitals showed that RADs patients manifested a significantly increased number of COVID-19 cases than that in the general population (3). Similarly, Italy, one of the European countries hardest hit by the outbreak, has a significantly higher prevalence of COVID-19 among the RADs population than in the general population (1). In England, a study involving 168,691 subjects adopted an index of age-standardized mortality rate (ASMR), and identified a higher risk of COVID-19 infection in RADs population (11). According to a nationwide cohort study from Denmark, RADs patients had an increased COVID-19 associated hospitalization rate, compare to 4.5 million general population (12).

**Table 1:** The incidence, hospitalization rates, and mortality of COVID-19 in RADs patients in studies from different nations (Shown in Figure 1).

**Author**	**Country**	**Study**	**Reference object**	***N***	**Cutoff date**	**Outcomes**	**Ratio**
Moradi et al.	Iran	Retrospective cohort study	Non-rheumatic patients	456	February 1, 2020–February 29, 2020	Lower prevalence	3.6% vs. 8.7%
Ye et al.	China	Retrospective cohort study	Non-rheumatic patients	2,326	January 13, 2020–March 15, 2020	Similar mortality	9.52% vs. 9.54%
Zhong et al.	China	Retrospective cohort study	Families without RADs patient	6,228	March 20, 2020 and March 30, 2020	Higher prevalence	63% vs. 34%
Haberman et al.	USA	Case series	General population	86	March 3, 2020–April 3, 2020	Similar hospitalization rates	16% vs. 26%
D'Silva et al.	USA	Retrospective cohort study	Non-rheumatic patients	52	January 30, 2020–April 8,2020	Similar hospitalization rates	44% vs. 40%
Pang et al.	China	Retrospective cohort study	General population	3,059	February 4, 2020–April 9, 2020	Higher prevalence	SLE: 163 per 100,000 vs. 27-70 per 100,000, RA: 490 per 100,000 vs. 280 per 100,000
Michelena et al.	Spain	Retrospective cohort study	General population	959	March 26, 2020–April 10, 2020	Similar prevalence	0.48% vs. 0.58%
Vázquez-Díaz et al.	Spain	Retrospective cohort study	General population	1,537	March 13, 2020–April 12, 2020	Similar prevalence and mortality	Prevalence: 0.49% vs. 0.5%, mortality 0.92% vs. 2%
Emmi et al.	Italy	Retrospective cohort study	General population,	458	April 1, 2020–April 14, 2020	Similar prevalence	0.22% vs. 0.2%
Pablos et al.	Spain	Retrospective cohort study	General population,	26,131	April 7, 2020–April 17, 2020	Higher prevalence	0.76% vs. 0.58%
Sarzi-Puttini et al.	Italy	Retrospective cohort study	General population	10,260	March 15, 2020–April 23, 2020	Similar prevalence	0.65% (RADs patients)
Ferri et al.	Italy	Retrospective cohort study	General population,	1,641	March 15, 2020–April 25, 2020	Higher prevalence	1.5% vs. 0.3%
Zen et al.	Italy	Retrospective cohort study	General population	916	April 9, 2020–April 25, 2020	Similar prevalence	0.21% (RADs patients)
Peach et al.	UK	Retrospective cohort study	General population,	168,691	March 1, 2020–April 30, 2020	Higher age-standardized mortality	1.38 fold (% NA)
Murray et al.	Ireland	Retrospective cohort study	General population	1,381	April 28, 2020–May 5, 2020	Similar prevalence	0.46% vs. 0.44%
Tan et al.	UK	Retrospective cohort study	RADs patients during influenza infection	133,589	January–June, 2020	Higher 30-day mortality	(2.2% to 4.3%) vs. (6.3% to 24.6%)
Nuño et al.	Spain	Retrospective cohort study	General population	122	February 25, 2020–June 8, 2020	Similar mortality	NA
Cordtz et al.	Denmark	Retrospective cohort study	General population	58,052	March 1, 2020–August 12, 2020	Higher hospitalization rates	HR 1.46
							95% CI 1.15-1.86 (% NA)

*RADs, rheumatic autoimmune diseases; Ratio, % of COVID-19 infected RADs patient versus (vs.) % of COVID-19 infected reference population or hazard ratio (HR); N, sample size; SLE, Systemic lupus erythematosus; RA, Rheumatoid arthritis; CI, confidence interval; NA, data not avaliable*.

However, studies have also shown that the diagnostic rate of SARS-CoV-2 in RADs patients does not necessarily increase, at least, they are less likely to develop into severe cases (13, 14). By analyzing the 2-month electronic medical records of Huoshenshan Hospital in Wuhan, China, a study showed the admission rate of ICU, hospital stays and mortality of COVID-19 in RADs patients were similar to the non-RADs patients (15), and these points have been supported by other cohort studies (16–18). It has even been reported that the prevalence of COVID-19 in RADs patients is lower than that of randomly selected non-RADs patients (19), which is based on the explanation that RADs patients are more consciously following the rules of epidemic prevention and control.

The proportion of each RAD in COVID-19 infected patients also varied. COVID-19 symptoms have been reported to be closely associated with rheumatoid arthritis (RA), vasculitis, spondyloarthrosis (SpA), psoriatic arthritis (PsA), and sicca syndrome (12, 13, 20, 21). Although, it has been suggested that the incidence of COVID-19 is higher in connective tissue diseases (CTDs) patients than those with inflammatory arthritis (IA) (1), we found that in most studies (4, 18, 22–25), RA accounts for a significantly higher proportion, with a range from 3.9 to 40.65% (Table 2). In addition to cases of COVID-19 infection in RADs patients, COVID-19 also appears to promote the development of RADs. During the COVID-19 outbreak, the prevalence of dermatomyositis was witnessed surge in India (26). Regarding the inconsistent prevalence of COVID-19 among distinct RADs, a study speculated that for certain RADs, the activity of underlying immune pathways were not completely the same (27), which results in the diverse involvement of RAD in the pathogenesis. Besides, this trend is also associated with the incidence of specific RADs themselves, thus it cannot be concluded that the prevalence of these diseases in COVID-19 is elevated.

**Table 2:** The proportion of different RADs in COVID-19 infection.

**Author**	**Country**	***N***	**RA (%)**	**SpA (%)**	**CTD (SLE included) (%)**	**PsA (%)**	**Vasculitis (%)**	**Polymyalgia rheumatica (%)**	**SS (%)**	**Miscellaneous (%)**
Nuño et al.	Spain	122	33.6	19.7	10.7	10.7	NA	NA	NA	25.4
Freites Nuñez et al.	Spain	123	40.65	14.63	6.5	4.88	1.63	NA	7.32	4.88
Scirè et al.	Italy	232	34.1	26.3	21.1	NA	11.2	NA	NA	NA
Tan et al.	UK	133,589	3.9–18.9	NA	NA	3.5–32.5	3.3–17.6	4.88	NA	NA
Montero et al.	Spain	62	32	26	36	NA	NA	NA	NA	NA
Galarza-Delgado et al.	Mexico	38	39.47	8.5	34.21	6.9	NA	NA	NA	NA
Gianfrancesco et al.	Australia	600	38	8	14	12	7	2	5	5

*RADs, rheumatic autoimmune diseases; N, patients with rheumatic immune diseases with COVID-19; RA, rheumatoid arthritis; SpA, spondyloarthritis; CTD, connective tissue diseases; SLE, systemic lupus erythematosus; PsA, psoriatic arthritis; SS, Sjogren Syndrome; NA, data not avaliable*.

Practically, these views are in dynamic change, and a number of meta-analyses and systematic reviews are in progress (International prospective register of systematic reviews, PROSPERO). In the second half of 2020, the COVID-19 pandemic has rebounded, which will certainly influence the evolution of disease.

## Influencing Factors of RADs Complicated With COVID-19

As with any disease, multiple factors can influence the infection and evolution of COVID-19 in RADs patients. Several factors that may be closely related to the disease were summarized from the existing studies.

### Age and Gender

**Figure 2:**
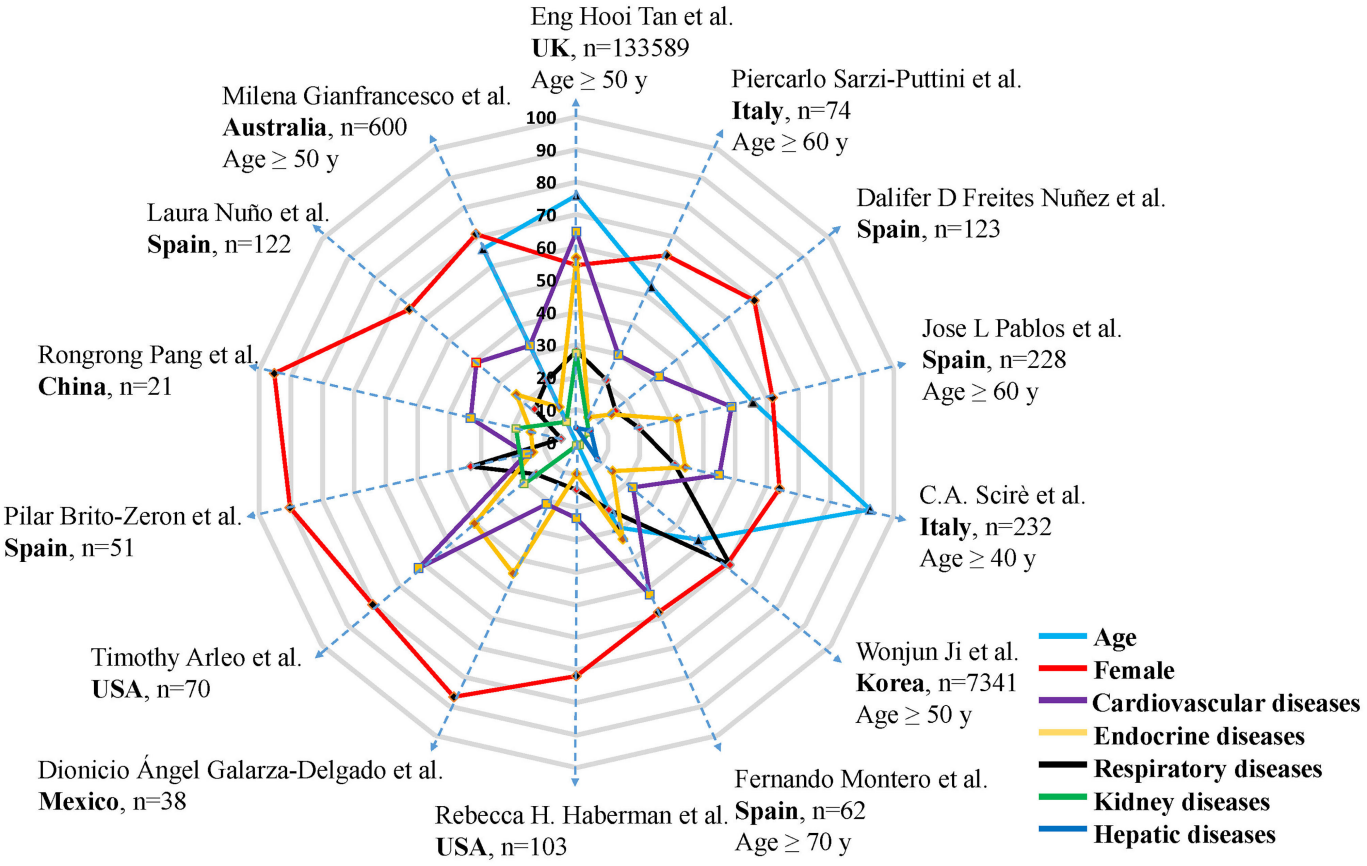
Ratio of female, elder patients, and comorbidity in RADs patients with COVID-19. A total of 14 articles were illustrated. Authors, country, sample size, different cut-off points of age were detailed in the figure. Each dotted blue line with arrows connects indicators from the same study, each dot represents one study, age (study *n* = 7), female (study *n* = 14), comorbidity (study *n* = 14). Different colors represent corresponding parameters, n represents the number of RADs patients infected with COVID-19. Each layer (gray line) on the radar map represents 10 percent.

It is generally accepted that patients diagnosed with COVID-19 tend to be elderly (5, 20, 28). Aging was seen as a factor independently related to hospital admission in RADs patients infected with SARS-CoV-2 (4). In many studies (5, 20, 22, 23, 25, 28, 29), cases over the age of 50 often account for more than half of all cases (Figure 2). But what is noteworthy is that the incidence of COVID-19 skewed toward a younger crowd gradually. Upon COVID-19 infection, age-specific mortality rates in RADs patients notably increased from the age of 35 years old, while in the uninfected population, this indicator began from the age of 55 years old (11).

Male and female patients behave differently in COVID-19 outbreak. The majority of RADs patients infected with COVID-19 were female (4, 5, 15, 18, 20, 22, 23, 25, 28–32), generally exceeding 60%, with an average of 70.86% (Figure 2). The overall mortality during COVID-19 development was higher in female RADs patients (11), which is different from the situation in non-RAD population that male accounts for a greater proportion. However, there were also other studies reporting that male could be an independent factor associated with the severity of COVID-19, for male RADs patients may have worse prognosis, higher hospitalization rate and risk of severe pneumonia (22, 28).

### Comorbidities

More unfavorable effects tend to occur when RADs patients suffer from comorbidities, especially under the condition of COVID-19 infection. In view of this, during the COVID-19 epidemic, rheumatologists paid considerable attention to the adverse effects of comorbidities on RADs patients. Overall, after data integration of 14 articles retrieved (4, 5, 15, 18, 20, 22–25, 28–32), the most common comorbidities in RADs patients infected with COVID-19 were cardiovascular diseases (37.34%), endocrine diseases (25.02%) and respiratory diseases (23.45%) (Figure 2), which were mainly attributed to hypertension, diabetes, chronic obstructive pulmonary disease and asthma, respectively. Renal diseases and hepatic diseases also account for a small proportion (11.57 and 6.14%, respectively). In the COVID-19 pandemic, the number of comorbidities are strongly associated with the general condition of patients, comprehensive recognition of the complexity of the diseases is of great significance. RADs patients with coexisting comorbidities should be classified for treatment in order to develop an ideal treatment plan.

### Race and Geography

Among different races, due to the influence of genetics, survival environment or life styles, slight or apparent discrepancies exist in the disease manifestations. The proportion of RADs patients from different ethnic groups varies in the COVID-19 pandemic (30, 31, 33, 34). In a race-focused study on 1,324 patients, by using multivariable models, researchers compared the clinical manifestations among African American, Latinx, Asian, and White patients. Results showed that RADs patients of the three former races have higher COVID-19 associated hospitalization rates than White patients, and the need for ventilator support is greater among Latinx patients, but no statistical difference in the mortality of COVID-19 was observed (35).

Geographical factors may affect the spread and evolution of COVID-19. As we mentioned in the epidemiological features section, studies from different countries or regions revealed disparities in morbidity, hospitalization rate or mortality of COVID-19 in RADs patients (1–3, 13, 14, 36), and ultimately affect the overall management and prevention of the COVID-19 pandemic. Mehta et al. (37) noted geographic differences in cognition on disease nursing, therapy adjustment and follow-up, especially in areas with a high COVID-19 incidence.

## Treatments

Formulating an effective therapeutic regimen is central to combat COVID-19, rheumatologists have made many attempts to address this problem in RADs patients. Amongst, the common drugs mainly include glucocorticoid, conventional disease modifying anti-rheumatic drugs (csDMARDs), antimalarial drugs and biologic or targeted synthetic disease-modifying anti-rheumatic drugs (b/tsDMARDs). Either maintenance treatment prior to diagnosis of COVID-19 or treatment initiated after diagnosis, these drugs act on COVID-19 infected RADs patients based on different pharmacological mechanisms, and have their own merits or limitations.

### Corticosteroids

The role of glucocorticoids in the treatment of rheumatic diseases is of landmark significance, while its role in rheumatic patients with COVID-19 infection is controversial. Results from the retrospective (*n* = 600) or prospective (*n* = 103) study found that prednisone use caused an increased hospitalization rates, especially when the dose of prednisone exceeded 10 mg per day, and disease activity was not significantly associated with glucocorticoid-induced hospitalization rates. Compared with non-hospitalized patients, the proportion of SLE and vasculitis was higher in hospitalized patients, while the proportion of psoriatic arthritis and ankylosing spondylitis was lower (25, 38). Another study has also confirmed the dose-dependent effect of glucocorticoid on positive diagnostic rate as well as COVID-19 associated hospitalization rates (7). In addition, Nuño et al. (18) found an increased mortality after glucocorticoid treatment in RADs patients. What is noteworthy is that although, glucocorticoids use seems to be associated with an increased risk of COVID-19 infection, rheumatologists suggest that it is reasonable to reduce glucocorticoids gradually to 5–7.5 mg/day, discontinuation of glucocorticoids during the pandemic is not recommended (10). After all, in single case reports, improvements of renal, respiratory, neurological and liver function in systemic lupus erythematosus (SLE) or SLE-like patients infected with COVID-19 were observed (39, 40).

### Conventional Synthetic Disease-Modifying Anti-rheumatic Drugs

With multiple categories and unique mechanism, csDMARDs play a key role in the treatment for RADs. Taking csDMARDs has been reported to benefit clinical outcomes by reducing the risk of COVID-19 infection, the protective effect of leflunomide on the cytokine storm emerging in severe COVID-19 cases was also proposed (19, 41). In addition, RADs patients treated with cyclosporine A and tacrolimus during COVID-19 infection had a relatively mild clinical course and a reduced susceptibility to reinfection (42). A meta-analysis involving eight studies showed that colchicine reduced the mortality of COVID-19 patients and the number of severe cases, which could be good news for gout sufferers (43).

However, in the vast majority of cases, the effect of csDMARDs on COVID-19 infection was neutral. A joint multinational, retrospective study of Asia, European and North America utilized network data and found no extra risk of severe events on a 30-day combined use of hydroxychloroquine (HCQ) and sulfasalazine (44). Likewise, in RADs patients treated with csDMARDs alone (methotrexate or cyclosporine) or in conjunction with biological agents or Janus Kinase inhibitors, no influence on the susceptibility to COVID-19 or hospitalization rate was observed (25, 45). Based on the neutral roles of csDMARDs, tapering or even discontinue of csDMARDs are suggestive strategies to recover anti-infection immunity in severe cases, which may help eliminate the virus rapidly (46).

### Antimalarial Drugs

Based on the long history of safe and effective use in RADs (47), antimalarial drugs have received unprecedented attention in fighting against COVID-19. As one of the typical antimalarial drugs, the role of HCQ in RADs with COVID-19 was studied in many countries and regions. In a previously mentioned study, RADs patients who had taken HCQ were less susceptibility to COVID-19 compared to patients who had taken other csDMARDs (OR 0.09, *p* = 0.044) (2). And another retrospective study suggested that long-term treatment of HCQ in RADs patients (at least 2 g/month) can protect against COVID-19 infection (48).

However, there were also studies have proposed the conservative effect of HCQ in treatment for RADs patients infected with COVID-19. Among SLE patients, whether treated with HCQ or without, the infection of SARS-CoV-2 and clinical symptoms were comparable, implying that chronic HCQ therapy did not have satisfactory results in inhibiting COVID-19 progression (7). Besides, past exposure to HCQ also did not prevent disease progression (49), RADs patients treated with HCQ may still be affected by COVID-19 in severe cases (40, 49–51).

In general, there are a lot of controversies about HCQ efficacy in rheumatism associated COVID-19. Nonetheless, during COVID-19 epidemic, HCQ is proposed to be the only treatment that is likely to increase survival in SLE patients, and it is not advisable to discard the HCQ-included regimen. American College of Rheumatology's guideline suggests that it is reasonable to adjust HCQ dosing intervals or dosage according to the practical situations (52).

The incidence of HCQ associated adverse events (AEs) was affected by the length of hospital stay (53). The incidence of HCQ related AEs was not significant after 1 week of short-term use, whereas, increased after 2–4 weeks of treatment (54, 55). Notably, the majority of AEs tends to occur with the first cumulative dose of 1 g, after which the frequency of AEs declined (56).

### Biologic Or Targeted Synthetic Disease-Modifying Anti-rheumatic Drugs

Given their broad molecular mechanisms, b/tsDMARDs may be potentially useful in alleviating inflammatory cytokine storm under COVID-19 attack (57). Different b/tsDMARDs affect the COVID-19 course differently. Compared with RADs patients treated with CD20 monoclonal antibody rituximab or IL-17A antagonist secukinumab, patients receiving tumor necrosis factor (TNF) inhibitors etanercept and adamuzumab or IL-6 receptor antagonist tocilizumab may experience a relatively mild course (9, 18, 58). In addition, the effects of TNF inhibitors and tocilizumab on clinical epidemiology have also been reported. Patients receiving adalimumab or infliximab treatment had a reduced risk of COVID-19 infection (19), and monotherapy of TNF-inhibitor prior to COVID-19 infection may reduce the COVID-19-related hospitalization or severity (12, 25, 31, 36). Treatment of tocilizumab has been proven to ensure stability of vascular permeability and myocardial function in RA patients infected with COVID-19 (59, 60), and also reduce the COVID-19 associated mortality and levels of inflammatory indicators (61, 62).

Nevertheless, many studies have shown the ineffectiveness, or even negative effects of b/tSDMARDs in the COVID-19 pandemic. In a clinical observation of small sample size (*n* = 21), in patients with COVID-19 associated pneumonia, tocilizumab administration showed no positive effect on ICU admission or 7-day mortality rate, compared to those who did not use tocilizumab (63), and patients treated with rituximab had a relatively higher hospitalization rate (64).

For the treatment after the infection of COVID-19, in general, the majority of studies have reported that these treatments just have a neutral effect on RADs patients, the mortality or hospitalization rate of patients have not been particularly affected. In contrast to the possible positive effect of maintenance treatment before diagnosis of COVID-19, the use of biologic agents after COVID-19 diagnosis did not stop the disease from progressing (Table 3).

**Table 3:** Treatments on RADs patients with COVID-19.

**Author**	**Country**	**Study**	***N***	**Cut-off Date**	**Glucocorticoid**		**csDMARDs**		**Antimalarial**		**t/sDMARDs**	
					***n***	**Efficacy**	***n***	**Efficacy**	***n***	**Efficacy**	***n***	**Efficacy**
Yousaf et al.	USA	Retrospective cohort study	6,548	January 20, 2020–June 11, 2020		NA	98	Neutral		NA	58	Neutral
Mathian et al.	France	Case series	17	March 29, 2020–April 6, 2020		NA		NA	17	Can not stop the progression to severity		NA
Teh et al.	Malaysia	Case series	5	March 1, 2020		NA		NA	5	Severe course needing aggressive therapy		NA
Price et al.	USA	Retrospective cohort study	239	March 10, 2020–April 21, 2020		NA		NA		NA	153	Survival improved
Freites Nuñez et al.	Spain	Prospective cohort study	123	March 1, 2020–April 24, 2020	61	Neutral	77	Neutral	27	Neutral	17	Risk reduced
Galarza-Delgado et al.	México	Case series	38	July 2020–August 2020		NA	28	Neutral	16	Neutral		NA
Loarce-Martos et al.	Spain	Retrospective cohort study	13	February 1, 2020–May 26, 2020		NA		NA		NA	13	Severe cases increased
Nuño et al.	Spain	Retrospective cohort study	122	February 25, 2020–June 8, 2020	47	Mortality increased	47	Hospitalization rates increased	25	Neutral	20	Hospitalization rates increased
Cavagna et al.	Italy	Retrospective cohort study	53	Febrary 2020–April 28, 2020		NA	53	Neutral		NA		NA
Zurita et al.	Ecuador	Case series	5	March 3, 2020–May 21, 2020		NA		NA	5	Neutral		NA
Colaneri et al.	Italy	Retrospective cohort study	112	March 14, 2020–March 27, 2020		NA		NA		NA	21	Neutral
Haberman et al.	USA	Prospective cohort study	103	March 3, 2020–May 4, 2020	13	Hospitalization rates increased		NA		NA	73	Neutral
Cordtz et al.	Denmark	Retrospective cohort study	138	March 1, 2020–August 12, 2020		Neutral		NA		Neutral		Neutral
Jung et al.	Korea	Retrospective cohort study	2,066	Before May, 2020		NA		NA	649	Neutral		NA
Arleo et al.	USA	Retrospective cohort study	70	February 1, 2020–July 31, 2020		NA	13	Hospitalization rates increased		NA	10	Hospitalization rates decreased

*The studies presented mainly includes treatment regimens after COVID-19 diagnosis. N, numbers of RADs patients infected with COVID-19; n, the number of people who received treatment; NA, data not available; Neutral, there is no particularly beneficial or harmful clinical effect*.

To sum up, no definitely positive or negative effect of any therapy can be totally determined, personalized medicine, and flexible adjustment are of outstanding importance.

## Applicable Laboratory Indicators

During the evolution of COVID-19 infection, several laboratory indicators fluctuate due to the immune system imbalance caused by viral attack and the inherent immune disorders in RADs patients, some of which may be helpful in monitoring disease fluctuations. At present, the indicators specific for RADs with COVID-19 infection are inconclusive. Here, we enumerate some indicators that may be useful for clinical guidance.

Typically, as a series of immune disorders mediated by inflammatory mechanisms, RADs usually exhibit alterations in inflammatory markers. Tan C et al. found that the significant increase of C-reactive protein (CRP) may be a potential early predictor for the severity of COVID-19, for CRP changes precede the imaging findings (65). And cohort studies reported a significant correlation between poor prognosis and elevated serum ferritin, IL-6 and procalcitonin (15, 66, 67). In their meta-analysis, Hariyanto et al. (68) summarized the predictive roles of increased procalcitonin (≥0.065 ng/ml), CRP (≥33.55 mg/L), D-dimer (≥0.635 μg/L), LDH (≥263.5 U/L) and decreased albumin (≤38.85 g/L) in the clinical prognosis of COVID-19 (68).

In addition, the proportion or number of immune cells can also be altered. Severe COVID-19 infected cases are more likely to have decreased lymphocytes, increased leukocytes counts and thus elevated neutrophil-lymphocyte-ratio (NLR). Moreover, either the percentage or the number of helper, memory helper and regulatory T cells can be reduced in RADs patients infected with COVID-19 (69). Unfortunately, although, a receiver operating curve (ROC) analysis of data from Hasan Sadikin Lupus Registry (HSLR) suggested an optimal cut-off of NLR at 2.94 can obtain a satisfactory sensitivity and specificity of the survival of SLE patient (70), there is no ideal cut-off of NLR can be applied to monitor COVID-19 condition yet.

It is worth noting that RADs related autoantibodies may present among non-RAD severe COVID-19 cases (Table 4). Lupus anticoagulants are common in COVID-19 patients (71, 72), and a level of over 15 U/mL of a-CL IgG may be an independent risk factor for the severity of COVID-19 (73). The ANAs-positive rate in COVID-19 patients can be as high as 50%, although, there was no significant difference in ANAs level between severe and mild patients (74–76). Elevated anti-SSA/Ro antibodies can be observed in COVID-19 patients with severe respiratory failure (77).

**Table 4:** Autoantibodies are present in patients with non-rheumatic diseases infected with COVID-19.

**Author**	**Country**	**N**	**Study**	**Cutoff date**	**Female (%)**	**Age (mean ± SD)/median (range)**	**Autoantibody**	**% of patients**
Reyes Gil et al.	USA	68	Retrospective cohort study	March 1, 2020-April 30, 2020	49.65	57.61 ±17.02	Lupus anticoagulant	44
Gatto et al.	Italy	122	Retrospective cohort study	January 15, 2020-April 30, 2020	50.8	54.3 ± 19.3	Anti cardiolipin (a-CL) IgG/IgM	13.4/2.7
							Anti-beta 2 glycoprotein I (β2GPI) IgG/IgM	6.3/7.1
Pascolini et al.	Italy	33	Prospectively cohort study	March 30, 2020-May 10, 2020	48.4	70 (22–90)	Antinuclear antibodies (ANAs)	33
							a-CL IgG and/or IgM	24
							Anti-β2-glycoprotein antibodies IgG and/or IgM	9
Zhang et al.	China	19	Retrospective cohort study	February 23, 2020-March 31, 2020	47.4	65 (60–70)	Anti-phospholipid antibodies	52.6
Bowles et al.	UK	35	Case series	Before May, 2020	31	56.6 (18.6-83.4)	Lupus anticoagulant	91
Amezcua-Guerra et al.	Mexico	21	Case series	April 12, 2020-April 19, 2020	57	62 (54–67)	Antiphospholipid antibodies	57.14
Bertin et al.	France	56	Retrospective cohort study	NA	50	66.6 (17.8)	a-CL IgG/IgM	100
Zhou et al.	China	21	Retrospective cohort study	January 28, 2020-March 2, 2020	38	66.10 ± 13.94	Anti-SSA/Ro antibodies	20
							Anti-60 kDa SSA/Ro antibodies	25
							ANAs	50
Vlachoyiannopoulos et al.	Greece	29	Case series	Before May	27.6	64.2 (43–85)	ANAs	34.5
							p-ANCA	6.9
							c-ANCA	6.9
							a-CL	24.1
							Anti-β2GPI antibodies	34.5
							Anti-cyclic citrullinated peptide (CCP) antibodies	3.5
Fujii et al.	Japan	2	Case series	Before August			Anti-SSA/Ro antibodies	2

*N, numbers of COVID-19 patient; SD, standard deviations*.

In summary, the sensitivity and specificity of clinical indicators may be affected by comorbidities, and a single indicator often reflects physiological changes in multiple organ functions. Because of the special practicability, there is an urgent need to find indicators with high specificity to monitor disease state.

## Discussion

In this study, we proposed some reflections on the dilemma faced by RADs patients in the COVID-19 epidemic by reading up on the viewpoints from a large number of studies. By integrating data on epidemiology, influencing factors, treatments and laboratory indicators, we found that the prevalence of COVID-19 in RADs patients did not necessarily increase, female and elderly patients account for a large proportion of RADs patients infected with COVID-19. Treatment with b/tsDMARDs TNF-inhibitors or tocilizumab may have a clinical benefit in reducing COVID-19 related hospitalization or mortality. Inflammatory markers such as procalcitonin, CRP, D-dimer, LDH, IL-6, or NLR may be useful in the surveillance of COVID-19 infection.

Objectively speaking, the incidence of COVID-19 in a region or country is closely related to the local capacity for disease control and awareness of the disease among the public. Behavioral factors, including wearing masks, improving sanitation, or even stopping the use of immunosuppressants without a doctor's instruction, may have influenced the researchers' assessment of true infection rates. In particular, as the COVID-19 vaccine becomes available, how well the vaccine supply matches the local population, as well as the coverage of the vaccinated population, will affect the global progress of COVID-19. Identification of the influencing factors is a tricky issue because it is difficult to standardize factors in different studies. In fact, in the overall COVID-19 population, males are more common than females (78), and in the absence of COVID-19, females usually make up a larger proportion in most RADs, thus, it is difficult to determine the true gender-related incidence of COVID-19 in RADs patients without the information about gender composition in local RAD population.

Moreover, flexible management of treatment regimens in response to COVID-19 is critical for rheumatologists. Regarding the administration of glucocorticoids, since there has been a dose-dependent effect on disease progression in RADs patients infected with COVID-19, glucocorticoids should always be administrated prudently. For the clinical efficacy of csDMARDs, both positive and negative effects possess their own evidence. However, neglecting the disease category of RADs or the grade of disease activity may affect the clinical significance of studies. Then, personalized medicine was highlighted again for the occurrence of some unexpected circumstances, such as the adverse events of HCQ, which showed a large heterogeneity; and the conventional dose of HCQ used for RADs was not applicable for the therapy of COVID-19 infection (79), which will certainly need to be adjusted on a case-by-case basis. Importantly, these conditions occur not only in the use of HCQ but also in other immune-mediated therapies. Also, because of the emergency situation of the COVID-19 pandemic, the observation periods of most studies were not long enough. Previous treatment exposure needs to be taken into account when evaluating clinical efficacy, cumulative effects or overlapping effects of drugs cannot be ruled out. As for the biologic agents, only the efficacy of tocilizumab has been suggested in meta-analyses, whereas, the efficacy of TNF inhibitors has been reported in only a few cohort studies and has not yet been supported by high-level evidence.

Each RAD has its characteristic pathogenesis, the immune pathways involved are not completely consistent. As mentioned earlier, autoantibodies can also present in some severe COVID-19 patients with non-rheumatic disease, which may confuse physicians, especially non-rheumatoid specialists, in their assessment of the patient's primary disease. Therefore, the clinical diagnosis of rheumatic autoimmune diseases must be carried out with a rigorous attitude in the context of the epidemic.

There are limitations in this study. First, this study is not a systematic review, rigorous verification of the quality of the included literature is also necessary. Selection bias is inevitable, studies with statistically positive results are more likely to be published, which may obscure the clinical significance of the negative results. Additionally, there may also be bias in the quality control of the included literature. In this article, single case reports or studies without complete information were excluded. Unfortunately, prospective studies are still scarce. Existing retrospective articles or case series are either not large enough in sample size or did not address a specific topic of RADs. In these studies, due to the differences in design and analysis, the influence of heterogeneity on the results cannot be disregarded. Based on the collection of more data, our subsequent studies will continue to address the limitations of this study.

In conclusion, RADs patients are experiencing unprecedented challenges in COVID-19 epidemic. The incidence of COVID-19 in RADs patients is not necessarily increased, which needs to be further, confirmed by more global data. Because RADs are commonly seen in females, elderly, and patients with comorbidities, the effect of these conditions on COVID-19 should not be ignored. The use of biologic agents such as TNF- inhibitors or IL-6 receptor antagonists appears to be clinically beneficial, but more evidence is needed. In addition, some inflammatory markers that are routinely used to evaluate RADs may also be useful for disease surveillance in COVID-19.

## Author Contributions

LD and JZ are responsible for the study topic selection and the revision of manuscripts. YZ is responsible for investigation, data processing, and original draft writing. All authors contributed to the article and approved the submitted version.

## Conflict of Interest

The authors declare that the research was conducted in the absence of any commercial or financial relationships that could be construed as a potential conflict of interest.

## Publisher's Note

All claims expressed in this article are solely those of the authors and do not necessarily represent those of their affiliated organizations, or those of the publisher, the editors and the reviewers. Any product that may be evaluated in this article, or claim that may be made by its manufacturer, is not guaranteed or endorsed by the publisher.
